# Eight weeks of supplementation with a multi-ingredient weight loss product enhances body composition, reduces hip and waist girth, and increases energy levels in overweight men and women

**DOI:** 10.1186/1550-2783-10-22

**Published:** 2013-04-19

**Authors:** Hector L Lopez, Tim N Ziegenfuss, Jennifer E Hofheins, Scott M Habowski, Shawn M Arent, Joseph P Weir, Arny A Ferrando

**Affiliations:** 1 The Center for Applied Health Sciences, 4302 Allen Road, STE 120, Stow, OH, 44224, USA; 2 Department of Exercise Science and Sport Studies, Rutgers University, New Brunswick, NJ, 08901, USA; 3 Department of Health, Sport, and Exercise Sciences, University of Kansas, Lawrence, KS, 66045, USA; 4 Center for Translational Research in Aging and Longevity, University of Arkansas for Medical Sciences, 4305 W. Markham St Slot 806, Little Rock, AR, 72205, USA

**Keywords:** Dietary supplement, Raspberry ketone, Adipokine, Body composition, Fat loss, Capsaicin

## Abstract

**Background:**

Numerous natural products are marketed and sold claiming to decrease body weight and fat, but few undergo finished product-specific research demonstrating their safety and efficacy.

**Objective:**

To determine the safety and efficacy of a multi-ingredient supplement containing primarily raspberry ketone, caffeine, capsaicin, garlic, ginger and Citrus aurantium (Prograde Metabolism™ [METABO]) as an adjunct to an eight-week weight loss program.

**Methods:**

Using a randomized, placebo-controlled, double-blind design, 70 obese but otherwise healthy subjects were randomly assigned to METABO or a placebo and underwent 8 weeks of daily supplementation, a calorie restricted diet, and exercise training. Subjects were tested for changes in body composition, serum adipocytokines (adiponectin, resistin, leptin, TNF-α, IL-6) and markers of health including heart rate and blood pressure.

**Results:**

Of the 45 subjects who completed the study, significant differences were observed in: body weight (METABO -2.0% vs. placebo -0.5%, P < 0.01), fat mass (METABO -7.8 vs. placebo -2.8%, P < 0.001), lean mass (METABO +3.4% vs. placebo +0.8%, P < 0.03), waist girth (METABO -2.0% vs. placebo -0.2%, P < 0.0007), hip girth (METABO -1.7% vs. placebo -0.4%, P < 0.003), and energy levels per anchored visual analogue scale (VAS) (METABO +29.3% vs. placebo +5.1%, P < 0.04). During the first 4 weeks, effects/trends for maintaining elevated serum leptin (P < 0.03) and decreased serum resistin (P < 0.08) in the METABO group vs. placebo were also observed. No changes in systemic hemodynamics, clinical blood chemistries, adverse events, or dietary intake were noted between groups.

**Conclusions:**

METABO administration is a safe and effective adjunct to an eight-week diet and exercise weight loss program by augmenting improvements in body composition, waist and hip girth. Adherence to the eight-week weight loss program also led to beneficial changes in body fat in placebo. Ongoing studies to confirm these results and clarify the mechanisms (i.e., biochemical and neuroendocrine mediators) by which METABO exerts the observed salutary effects are being conducted.

## Introduction

Obesity, particularly central adiposity, has been increasingly cited as a major health issue in recent decades. Indeed, some of the leading causes of preventable death and disability, including heart disease, stroke, type 2 diabetes, degenerative joint disease, low back pain, and specific types of cancer are obesity-related [1]. In the United States, more than one-third of adults (35.7%) are obese [2]. Annual obesity-related medical costs in the United States were estimated to be as high as $147 billion in 2009 [3].

Excess body weight is also a major risk factor for the development of Metabolic Syndrome. Metabolic Syndrome is a constellation of medical disorders including hypertension, central adiposity, hyperglycemia and dyslipidemia [4,5] that increase the risk of premature cardiovascular disease. Adipocytokines (including leptin, tumor necrosis factor-α, interleukin-6, resistin, visfatin, retinol binding protein-4, angiotensinogen and adiponectin) are signaling cytokines produced by adipose tissue. Adipose tissue acts as an endocrine organ producing adipocytokines to regulate insulin signaling, vascular tone, carbohydrate and lipid metabolism, and the inflammatory response. Dysregulation of certain adipocytokines can contribute to insulin resistance, amplified systemic inflammation and lead to the development of Metabolic Syndrome and hypertension [6]. For example, plasma levels of adiponectin have been reported to be significantly reduced in obese humans [7] and in patients with type-2 diabetes mellitus, hypertension and metabolic syndrome [8-11].

Alternative methods to aid weight loss include meal replacement preparations, and nutritional supplements such as vitamins, mineral, and botanicals. Raspberry ketone is an ingredient found in raspberries (*Rubus idaeus*) that may have weight loss potential given preliminary findings in rodents and cell cultures, i.e. prevention of weight gain during a high-fat diet, and enhanced norepinephrine-lipolysis, increased adiponectin expression, and translocation of hormone-sensitive lipase in adipocytes [12,13]. To date, however, the effects of raspberry ketone in humans remain unexplored. Many weight loss supplements include caffeine and capsaicin since they are known to increase energy expenditure by up to 13% and have been proposed to counteract the decrease in metabolic rate that often accompanies weight loss [14]. In humans, oral ingestion of certain capsaicinoids, (active component of chilli peppers from the genus *Capsicum*) has been shown to increase energy expenditure, lipolysis and fat oxidation [15], activate brown adipose tissue [16] and stimulate the systemic release of norepinephrine [15,17]. Bioactive compounds found in the rhizomes of ginger (*Zingiber officinale*) and garlic (*Allium sativum*) extracts have been shown to influence many key features of the metabolic syndrome by modulating adipocytokine secretion from adipose tissue, reducing body fat accumulation, decreasing circulating insulin and markers of systemic inflammation in murine and cell culture models, with similar findings emerging from studies in humans [18-21]. Extracts of Citrus aurantium, standardized for p-synephrine and other bioactive amines have been shown to increase resting metabolic rate and enhance weight loss in human clinical trials [22].

Prograde Metabolism™ (METABO) is a multi-ingredient dietary supplement that contains primarily raspberry ketone, caffeine, capsaicin, garlic, ginger and Citrus aurantium and is suggested to be used in combination with an exercise and nutrition program. The purpose of this study was to determine the safety and efficacy of METABO as an adjunct to an 8-week weight loss program. Primary endpoints included determination of the effect of this product on body composition and various anthropometric measures. Secondary endpoints included determination of safety information via measurement of systemic hemodynamics and standard clinical chemistry panels of sera and plasma.

## Methods

### Subjects

A total of 70 recreationally active males and females between the ages of 21 and 45 years were recruited to participate in the study. In this study, recreationally active was defined as participating in less than or equal to two exercise sessions (aerobic or anaerobic activity) per week over the previous 30 days. Subjects were required to have a body mass index (BMI) greater than 27 kg/m^2^ and body fat greater than 20% (for males) or greater than 25% (for females). Subjects were excluded if they had used weight-loss supplements within the 30 days prior to the start of the study, had gained or lost more than 4.5 kg over the previous 30 days, were currently taking medications that alter insulin sensitivity, or were using lipid lowering or antihypertensive drugs. Subjects were also excluded if they had metabolic disorders, heart disease, hypertension, a known allergy to any ingredients in the supplement or placebo or had smoked cigarettes in the last six months. Prior to being enrolled in the study, all subjects underwent a physical examination by a licensed physician, 12-lead electrocardiogram, health history screen and provided written informed consent. All procedures were approved by an independent Institutional Review Board (IntegReview, Austin, TX; protocol # PRO-002, approved 09/16/2011) and were conducted in accordance with the revised Declaration of Helsinki (2008).

### Experimental design

**Figure 1:**
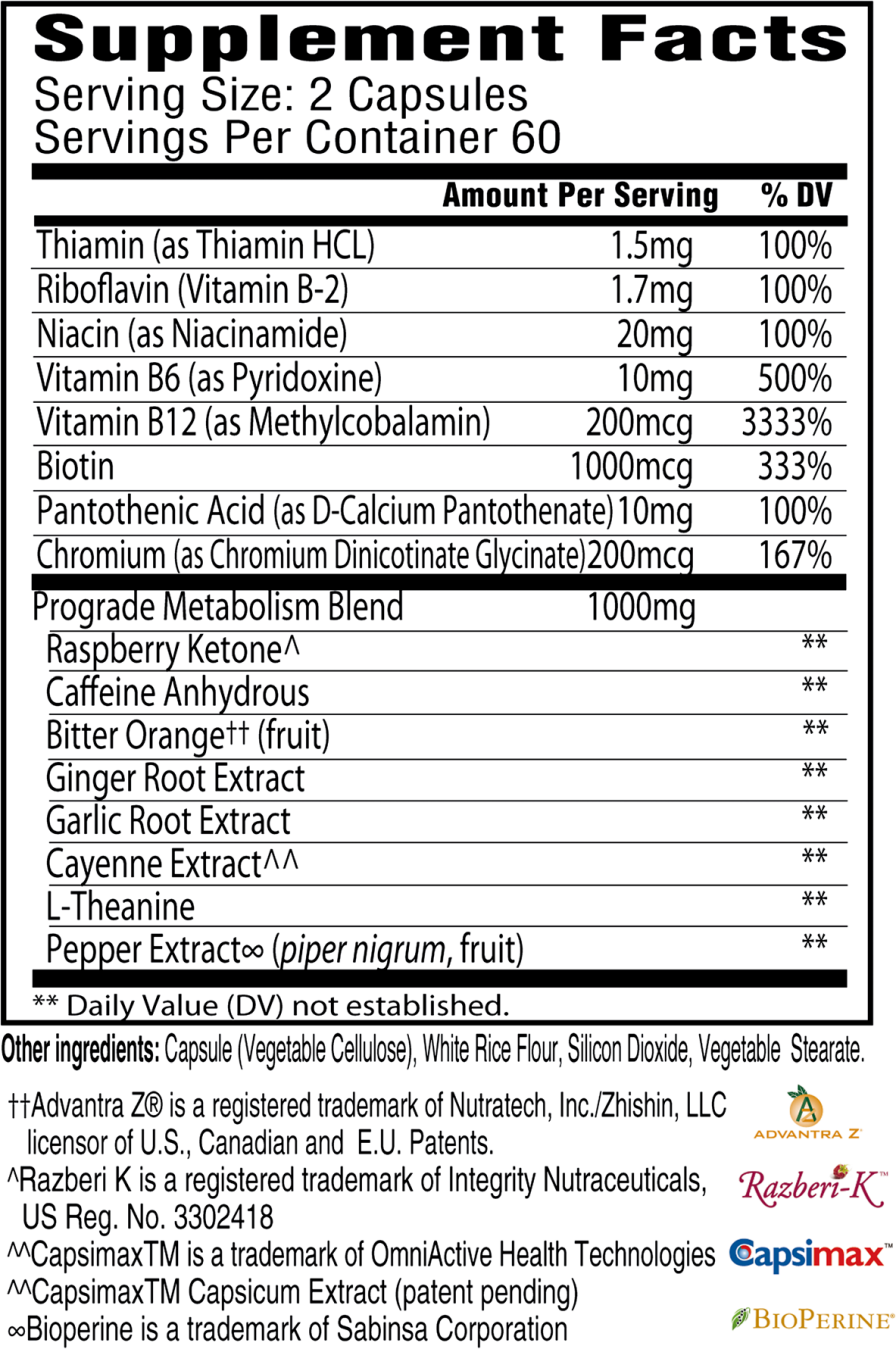
Supplement Facts panel for METABO.

This study utilized a randomized, placebo-controlled, parallel-group, double-blind design. Subjects were matched according to sex and BMI prior to being randomized into placebo or METABO groups. The placebo consisted of rice flour while the main ingredients of the METABO formula included raspberry ketone, caffeine, capsaicin, garlic organosulfur compounds, gingerols, shogaols, Citrus aurantium and related alkaloids, B vitamins, and chromium (see Figure 1 for the Supplement Facts panel). Capsules were produced in accordance with current Good Manufacturing Practices (cGMP) in a United States Food and Drug Administration (FDA) registered facility. Prior to production, all raw materials were tested for purity and potency. A sample of the lot and batch from the placebo and METABO finished product was tested by an independent third party for label claim and was shown to be within +/- 1% to 4.3% of the actual formulation for the main bioactive ingredients (Eurofins Scientific Inc., Petaluma, CA; Samples: #740-2011-00007867 and #740-2011-00007868).

The study intervention included an 8-week diet and exercise program consisting of recreationally active men and women, randomly assigned to receive either a placebo or the manufacturer recommended dosage of their respective supplement (two capsules with breakfast and two capsules with lunch). Prior to starting the study, a state-licensed, registered dietitian designed a target diet to provide approximately 500 kilocalories per day less than each subject’s estimated daily caloric requirement as estimated by the Mifflin-St. Jeor equation [23] x an activity factor of 1.2. In an effort to decrease variability, the 500 kcal deficit was prescribed consistently to every subject based on estimated energy expenditures from the Mifflin-St. Jeor equation, as opposed to targeting the 500 kcal deficit to their baseline 3-day diet records. Each subject was given seven days of menus based off their daily allowance for calories. All menus consisted of three meals and two snacks and targeted a 40% carbohydrate, 30% protein and 30% fat eating plan. Each study participant was contacted on a weekly basis to assess compliance to diet and supplement protocol. Subjects performed three, 60-minute exercise sessions per week using a ‘boot camp’ type of training. A typical class consisted of the following format: 10 minute warm-up (i.e. walking, light jogging, or biking); 30 minutes of circuit training (upper and lower body each session) composed of the following exercises: mountain climbers, squat thrusts, jumping jacks, squat kickouts, walking lunges, push-ups, dips, resistance band elbow flexion, extension and shoulder presses; 10 minutes abdominals/core, and 10 minutes cool down/stretching. Based on pilot data monitoring heart rate, this type of training expends approximately 300-400 kcal/session. Every training session was supervised by a certified fitness professional and conducted at a single local facility to verify participation, and all subjects trained as one group. The fitness professional used a participant attendance log to monitor training compliance. All subjects had measurements of their weight, BMI, waist and hip girths, body fat and lean mass taken at week 0 (baseline), week 4 (midpoint of the study) and week 8 (end of the study). A member of the research staff contacted all subjects on a weekly basis to ensure compliance to the supplementation protocol, and pill counts were performed during mid and post testing.

Blood samples were drawn at week 0 and week 8 for standard assessment of clinical laboratory parameters (i.e. comprehensive metabolic panel, lipid panel) and at weeks 0, 4 and 8 for serum concentrations of adipocytokines (adiponectin, resistin, leptin, tumor necrosis factor-α (TNF-α) and interleukin-6 (IL-6)). Vital signs, including blood pressure and heart rate, were also recorded at weeks 0, 4 and 8. For each laboratory session, subjects reported to the laboratory normally hydrated (*ad libitum* water intake recorded prior to baseline testing and repeated prior to week 4 and week 8 testing), 12 hours postprandial and at least 48-hours following their last exercise session. All measurements were completed by the same researcher to minimize between-trial variation.

Energy levels and food craving data were analyzed using a whole unit Likert-type scale [24]. Food craving was defined as “an intense desire for a specific food that is difficult to resist.” Subjects rated the frequency of cravings ranging from 1 (“not at all”) to 5 (“nearly every day”). The Food Craving Inventory consists of five factors or scales measuring cravings for Sweets, Fast Food Fats, Fats (High Fats), Carbs (carbohydrates/starches) and Healthy foods [24]. All energy levels and food craving data were collected at weeks 0, 4 and 8.

### Dependent variables

Sera and plasma variables were measured from 20 mL (10 mL for sera and 10 mL for plasma) of blood drawn with stasis via venipuncture of an antecubital vein. All blood samples were taken in the morning at approximately the same time of day (i.e., between 0600 and 1000 h for all subjects, ± 60 min window of their initial visit) to minimize diurnal variation, and subjects used their target dietary recommendations (pre-intervention) to standardize their evening meal, including fluid intake, before mid (week 4) and post (week 8) testing. Blood samples were harvested into 10 mL into BD Vacutainer ® tubes with and without EDTA, chilled on ice for 15 minutes, and then centrifuged (Drucker Model 614, Philipsburg, PA) at room temperature for 15 minutes at 1200 × *g* to obtain plasma and serum, and immediately placed into two aliquots. One aliquot was immediately analyzed for a 21-item clinical chemistry profile (Hitachi D2400, Roche Diagnostics, Germany) by a certified clinical laboratory. This profile consisted of a comprehensive metabolic panel (glucose, BUN, creatinine, sodium, potassium, chloride, carbon dioxide, calcium, total protein, albumin, globulin, total bilirubin, alkaline phosphatase, AST [SGOT], and ALT [SGPT]) as well as a lipid profile (total cholesterol, HDL-C, LDL-C, VLDL-C, triacylglycerols [TAG]). The second aliquot was stored at -80°C until later batch analysis for serum adipokines (adiponectin, resistin, leptin, TNF-α, IL-6) via enzyme-linked immunosorbent assay. Adipokines were analyzed using a MAGPIX® (Luminex Corporation, Austin, TX) and customized commercially available magnetic bead panels (Millipore Corporation, Billerica, MA). Adiponectin and resistin were analyzed with a Human Adipokine Magnetic Bead Panel 1 (Millipore catalog # HADK1MAG-61 K), while IL-6, TNF-alpha, and leptin were analyzed with a Human Adipokine Magnetic Bead Panel 2 (Millipore catalog # HADK2MAG-61 K). Prior to each assay, the MAGPIX was calibrated using the MAGPIX Calibration Kit (Millipore catalog # 40-049) and performance verified using the MAGPIX Performance Verification Kit (Millipore catalog # 40-050). Each assay was run in one batch, therefore no inter-assay CV was determined. Intra-assay CV was 4% for adiponectin and 3% for resistin, while CVs for IL-6, TNF-alpha, and leptin were 2%, 3%, and 5%, respectively.

Body weight and height were determined on a calibrated Seca 767™ Medical Scale and a wall-mounted stadiometer, respectively. Body mass index was calculated as: BMI = (weight in kg)/(height in m^2^). Lean mass, percent body fat and trunk limb fat ratios were assessed using dual energy x-ray absorptiometry (DEXA, GE Lunar, DPX Pro). All DEXA scans were performed by the same technician and analyzed via current manufacturer software (enCORE version 13.31). Female subjects were measured during the early follicular phase of their menstrual cycle, based on reported last menstrual period, to minimize effects of menstrual hormonal changes on dependent variables. Briefly, subjects were positioned in the scanner according to standard procedures and remained motionless for approximately 15 minutes during scanning. DEXA segments for the arms, legs, and trunk were subsequently obtained using standard anatomical landmarks. Percent fat was calculated by dividing fat mass by the total scanned mass. Quality control calibration procedures were performed prior to all scans using a calibration block provided by the manufacturer. Prior to this study, we determined test-retest reliability for repeated measurements of lean mass, bone mineral content, and fat mass with this DEXA via intra-class correlation coefficients [25]. All values were > 0.98.

Waist girth (defined as the narrowest part of the trunk between the bottom of the rib cage and the top of the pelvis) and hip girth (defined as the largest laterally projecting prominence of the pelvis or pelvic region from the waist to the thigh) were measured in duplicate using standardized anthropometric procedures [26]. Seated, resting heart rate and blood pressure were measured in duplicate using an automated sphygmomanometer (Omron HEM-711).

A baseline 3-d food record was completed for each subject after screening and enrollment, prior to randomization and intervention. To verify dietary compliance, subjects completed 3-d food records (which included two weekdays and one weekend day) during baseline testing, week 4, and week 8. All food records were analyzed by a state-licensed, registered dietitian using commercially available software (NutriBase IV Clinical Edition, AZ). To enhance accuracy of the food records, all subjects received instruction during baseline testing on how to accurately estimate portion sizes. This counseling was reinforced during each visit to the laboratory. No other dietary supplements were allowed with the exception of standard strength multivitamins.

### Safety analysis

Safety and tolerability of the supplements were assessed through adverse event reports that were coded using the Medical Dictionary for Regulatory Activities (MedDRA). The intensity of an adverse event was graded according to the protocol-defined toxicity criteria based on the 2009 DAIDS Therapeutic Research Program’s “Table for Grading Severity of Adult Adverse Experiences [27].”

### Statistical analyses

Descriptive data are summarized using mean ± standard deviation (SD). Differences between groups from baseline to week 4 and baseline to week 8 were analyzed using analysis of covariance (ANCOVA) with the baseline scores employed as the covariate. All analyses were verified to meet the homogeneity of regression assumption (parallelism) of ANCOVA. Non-normally distributed variables were log-transformed before analysis. For descriptive purposes, raw values as well as the change scores (week 4 minus baseline, week 8 minus baseline) of all dependent variables are displayed. Statistical significance was accepted when the probability of a type 1 error was less than or equal to 0.05 (p ≤ 0.05). Data were analyzed using statistical software written using LabVIEW (National Instruments, Austin TX) programming software. Our *a priori* power analysis indicated that approximately 42 total subjects were required to have an 80% chance of detecting, at the 5% level of significance, a difference between the two groups in body fat mass of 3 kg. However, a goal of 70 total subjects were be enrolled due to higher expected attrition from a study involving multiple independent variables including a prescribed diet, regular exercise, and supplement intervention.

## Results

### Subjects

Of the 70 subjects initially recruited, 25 were lost due to attrition (i.e., poor compliance to the diet [n = 11], supplement regimen [n = 12], exercise program [n = 7], request to withdraw [4], and pregnancy [n = 1]). Of the 45 subjects who completed the study, the group who received placebo consisted of n = 18, seven male and 11 female subjects. The group who received METABO consisted of n = 27, 12 male and 15 female subjects. Subject demographics were similar between the two groups (Table 1).

**Table 1:** Baseline characteristics of subjects

**Variable**	**Placebo (n = 18)**	**METABO (n = 27)**
Age (y)	34.9 ± 5.7	35.9 ± 5.9
Height (m)	1.72 ± 0.1	1.73 ± 0.1
Body mass (kg)	91.0 ± 25.1	94.3 ± 23.3
BMI (kg/m^2^)	30.8 ± 2.5	31.5 ± 2.3
Fat mass (kg)	32.56 ± 13.5	37.18 ± 14.9
Lean mass (kg)	50.47 ± 13.6	52.81 ± 13.5
Lean:fat ratio	1.78 ± 0.77	1.61 ± 0.65
Waist (cm)	104.6 ± 18.3	104.1 ± 15.3
Hip (cm)	113.6 ± 15.1	114.3 ± 13.4
Waist:hip ratio	0.92 ± 0.16	0.91 ± 0.13
Systolic BP (mm Hg)	119 ± 11	120 ± 10
Diastolic BP (mm Hg)	80 ± 5	78 ± 9
Resting HR (beats/min)	69 ± 8	70 ± 8
Fasting glucose (mg/dL)	91 ± 8	90 ± 8

Values are mean ± SD.

Key: *BMI*, body mass index (body mass in kg/height in m^2^), *BP*, blood pressure, *HR*, heart rate.

### Anthropometric variables

**Figure 2:**
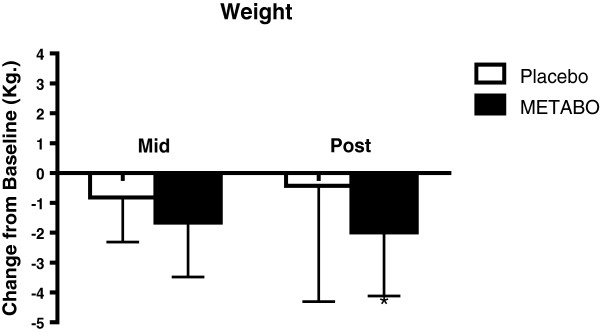
**Mean ± SD changes in body weight, relative-to-baseline, in subjects who received METABO and placebo.** * indicates statistically significant difference (P < 0.05) between groups during the post time point via ANCOVA.

**Figure 3:**
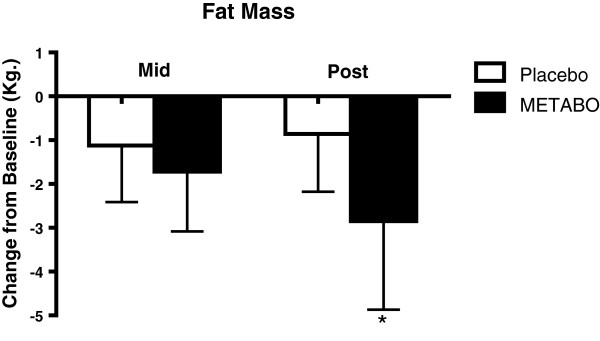
**Mean ± SD changes in body fat mass, relative-to-baseline, in subjects who received METABO and placebo.** * indicates statistically significant difference (P < 0.05) between groups at the post time point via ANCOVA.

**Figure 4:**
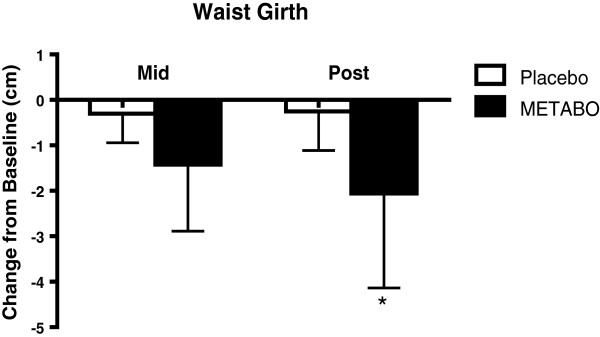
**Mean ± SD changes in waist girth, relative-to-baseline, in subjects who received METABO and placebo.** * indicates statistically significant difference (P < 0.05) between groups at the post time point via ANCOVA.

**Figure 5:**
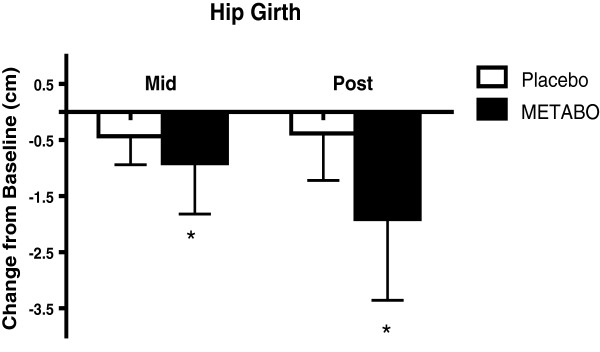
**Mean ± SD changes in hip girth, relative-to-baseline, in subjects who received METABO and placebo.** * indicates statistically significant difference (P < 0.05) between groups at the mid and post time points via ANCOVA.

**Figure 6:**
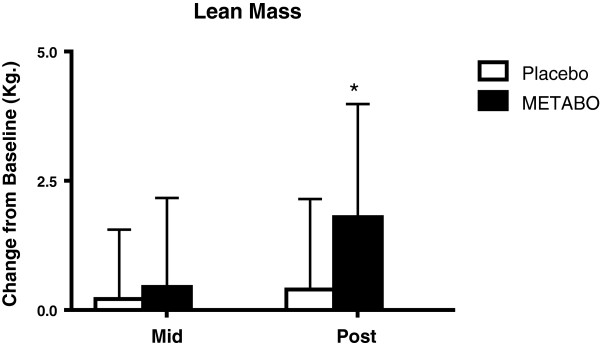
**Mean ± SD changes in lean body mass, relative-to-baseline, in subjects who received METABO and placebo.** * indicates statistically significant difference (P < 0.05) between groups at the post time point via ANCOVA.

**Figure 7:**
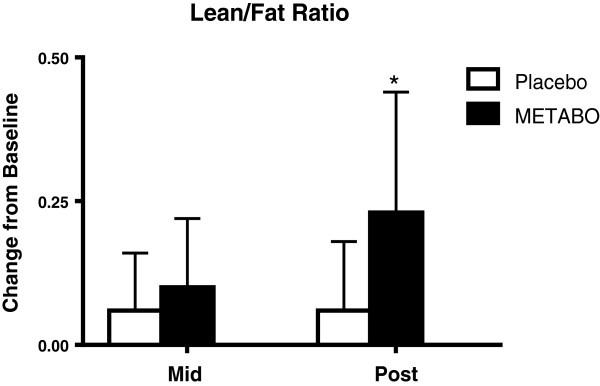
**Mean ± SD changes in lean mass-to-fat mass ratio, relative-to-baseline, in subjects who received METABO and placebo.** * indicates statistically significant difference (P < 0.05) between groups at the post time point via ANCOVA.

Anthropometric variables are presented in Table 2. Statistically significant decreases were observed from week 0 to week 8 for subjects who received METABO versus those who received placebo in body weight (-2.0% versus - 0.5%; p < 0.01, Figure 2); fat mass (-7.8% versus -2.8%; p < 0.001, Figure 3); waist girth (-2.0% versus -0.2%; p < 0.0007, Figure 4) and hip girth (-1.7% versus -0.4%; p < 0.0003, Figure 5). Subjects who received METABO exhibited statistically significant increases in lean mass compared to those who received placebo (+3.4% versus +0.8%; p < 0.03, Figure 6). The lean/fat ratio of subjects who received METABO also increased significantly more (+14.3%) compared to subjects who received placebo (+3.4%, p < 0.001, Figure 7).

**Table 2:** Anthropometric variables of METABO and placebo groups from week 0 through week 8

**Variable**	**METABO**			**Placebo**			**P**
	**n = 27**			**n = 18**			**Value**^**1**^
	**Baseline**	**Mid point**	**End of study**	**Baseline**	**Mid point**	**End of study**	
	**(Week 0)**	**(Week 4)**	**(Week 8)**	**(Week 0)**	**(Week 4)**	**(Week 8)**	
Body weight (kg)	94.1 ± 23.3	92.5 ± 23.1	92.2 ± 23.3	90.7 ± 25.1	90.1 ± 24.7	90.3 ± 24.8	0.10, 0.01*
Fat mass (kg)	37.2 ± 14.9	35.5 ± 14.7	34.3 ± 14.8	32.6 ± 13.5	31.4 ± 12.7	31.7 ± 12.7	0.16, 0.001*
Lean mass (kg)	52.8 ± 13.5	53.3 ± 14.1	54.6 ± 13.8	50.5 ± 13.6	50.7 ± 13.8	50.9 ± 13.6	0.72, 0.03*
Waist (cm)	104.1 ± 15.3	102.7 ± 15.1	102.0 ± 14.7	104.6 ± 18.3	104.2 ± 15.1	104.3 ± 18.1	0.004*, 0.0007*
Hip (cm)	114.3 ± 13.4	113.4 ± 13.2	112.4 ± 13.5	113.6 ± 15.1	113.2 ± 14.9	113.2 ± 14.9	0.04*, 0.0003*

Values are mean ± SD.

^1^P values are for the differences between the two groups, METABO versus placebo.

*Significant result P < 0.05 via ANCOVA (i.e., week 4 and week 8 time points are significantly different from each other after using the week 0 time point as the covariate).

From week 0 to week 4 the mean differences in decreased waist girths for the subjects who received METABO versus those who received placebo were -1.36% and -0.4%, respectively, and the differences between groups were statistically significant (p < 0.004). Similarly, the mean differences in decreased hip girths for the subjects who received METABO versus those who received placebo were -0.8% and -0.4%, respectively, and were statistically significant (p < 0.045). However, from week 0 to week 4 there were no statistically significant differences in body weight (p < 0.11), fat mass (p < 0.18), or lean mass (p < 0.72) between groups.

### Dietary variables

The macronutrient intake for METABO and placebo groups, at baseline (pre-intervention) and at the end of study based on 3-day food records is summarized in Table 3. The mean target daily dietary intake (calculated using the Mifflin-St. Jeor equation x 1.2 activity factor – 500 kcals) for METABO was 1955 kcal, 195 g carbohydrates, 147 g protein, and 87 g of fat. The target intake for placebo was 1907 kcal, 191 g carbohydrates, 143 g of protein, and 85 g of fat. No differences were observed in energy consumption, or in absolute or relative amounts of dietary carbohydrate, protein or fat between METABO and placebo.

**Table 3:** Dietary intake of METABO and placebo groups from week 0 through week 8 using 3-day food records

**Variable**	**METABO**			**Placebo**			**P**
	**n = 27**			**n = 18**			**Value**^**1**^
	**(Baseline) Pre-intervention**	**Mid point**	**End of study**	**(Baseline) Pre-intervention**	**Mid point**	**End of study**	
	**(Week 0)**	**(Week 4)**	**(Week 8)**	**(Week 0)**	**(Week 4)**	**(Week 8)**	
Energy (kcal/d)	1831 ± 491	1889 ± 428	1912 ± 423	1764 ± 482	1913 ± 432	1917 ± 479	0.48, 0.41
Carbohydrate (g/d)	206 ± 78	188 ± 58	188 ± 57	215 ± 94	191 ± 58	202 ± 61	0.94, 0.80
Carbohydrate (%)	46 ± 14	39 ± 6	39 ± 5	48 ± 15	40 ± 6	42 ± 5	0.70, 0.90
Fat (g/d)	54 ± 20	56 ± 17	57 ± 15	52 ± 23	57 ± 13	56 ± 13	0.87, 0.85
Fat (%)	26 ± 7	27 ± 4	27 ± 4	27 ± 10	27 ± 4	27 ± 4	0.98, 0.79
Protein (g/d)	130 ± 66	158 ± 43	162 ± 47	110 ± 50	161 ± 47	150 ± 50	0.77, 0.66
Protein (%)	28 ± 12	34 ± 8	34 ± 7	26 ± 13	34 ± 7	31 ± 6	0.52, 0.99

Values are mean ± SD.

^1^P values are for the differences between the two groups, METABO versus placebo at week 4 and week 8, respectively. No significant between group differences at week 4 or week 8 time points were noted using ANCOVA (where the week 0 time points were used as the covariate). Target dietary intake was provided to each subject after baseline 3-day food records (pre-intervention) using the Mifflin-St. Jeor equation plus an activity factor of 1.2 minus 500 kcal/day, with a macronutrient ratio of 40% carbohydrate, 30% fat and 30% protein.

### Metabolic variables

The effects of the diet + exercise + supplement regimen on metabolic characteristics are shown in Table 4. For all the blood lipids analyzed, cholesterol, HDL, LDL, cholesterol/HDL ratio and TAG, baseline levels in both groups were within normal ranges and did not significantly differ between them. Blood glucose increased slightly in both groups from week 0 to week 8 but these differences were not statistically significant (p < 0.60).

**Table 4:** Metabolic variables of METABO and placebo groups from week 0 through week 8

**Blood lipid**		**METABO**				**Placebo**			**P**
		**n = 27**				**n = 18**			**Value**^**1**^
	**Baseline**	**Mid point**	**End of study**	**%**	**Baseline**	**Mid point**	**End of study**	**%**	
	**(Week 0)**	**(Week 4)**	**(Week 8)**	**Change**	**(Week 0)**	**(Week 4)**	**(Week 8)**	**Change**	
Cholesterol, (mg/dL)	178.33 ± 26.49	NP	173.30 ± 30.25	-2.8	175.78 ± 31.45	NP	176.50 ± 31.14	0.4	0.3
HDL (mg/dL)	48.44 ± 12.47	NP	48.56 ± 15.26	0.2	50.28 ± 10.86	NP	48.94 ± 12.06	-2.7	0.49
LDL (mg/dL)	103.96 ± 26.04	NP	103.00 ± 30.92	-0.9	100.94 ± 28.53	NP	100.78 ± 30.17	-0.1	0.88
Cholesterol: HDL Ratio	3.91 ± 1.15	NP	3.85 ± 1.24	-1.5	3.67 ± 1.16	NP	3.87 ± 1.44	1.2	0.15
TAG (mg/dL)	118.44 ± 40.42	NP	99.59 ± 44.77	-15.9	120.22 ± 67.45	NP	117.06 ± 63.39	-2.6	0.07
Glucose (mg/dL)	89.81 ± 8.04	NP	92.67 ± 7.74	3.2	90.56 ± 8.3	NP	94.56 ± 13.82	4.4	0.60
Adiponectin (pg/mL)	10.20 ± 0.81	10.16 ± 0.74	9.93 ± 0.76	-0.2	10.17 ± 8.80	10.05 ± 0.80	10.04 ± 0.83	-0.3	0.47, 0.15
Resistin (pg/mL)	82.74 ± 38.47	81.65 ± 36.72	69.63 ± 26.04	-15.8	86.77 ± 50.18	68.38 ± 32.11	81.57 ± 46.75	-5.9	0.08, 0.26
Leptin (pg/mL)	8.99 ± 0.88	8.93 ± 0.94	8.729 ± 1.25	-3.0	8.85 ± 1.09	8.36 ± 1.07	8.76 ± 1.25	-3.0	0.03*, 0.5
lL-6 (pg/mL)	0.45 ±0.83	0.37 ± 0.56	0.34 ± 0.94	-24.5	0.45 ± 1.22	0.38 ± 0.82	0.38 ± 1.44	-14.8	0.97, 0.89
TNF-α (pg/mL)	1.71 ± 1.16	1.45 ± 1.04	1.58 ± 1.08	-7.6	1.35 ± 1.82	1.53 ± 1.67	1.19 ± 1.25	-11.7	0.41, 0.49

Values are mean ± SD.

^1^P values are for the differences between the two groups, METABO versus placebo at week 4 and week 8, respectively. No significant differences between the week 8 time points were noted using ANCOVA (where the week 0 time points were used as the covariate). *Significant difference at the week 4 mid time point for Leptin using ANCOVA. NP: not performed; HDL: high density lipoprotein; LDL: low density lipoprotein; TAG: triacylglycerols; IL-6: interleukin-6; TNF-α: tumor necrosis factor-α.

Concentrations of adipokine levels from week 0 to week 8 are also presented in Table 4. Serum leptin concentrations were not significantly different between the two groups from week 0 to week 8 but elevated serum concentrations of leptin were observed from week 0 to week 4 in METABO (p < 0.03) versus the placebo group. Resistin concentrations were normal in both groups and no significant treatment effects were observed, however decreased serum resistin concentrations from week 0 to week 4 approached significance (p < 0.08) for METABO. From week 0 to week 8 there were no differences in serum concentrations of adiponectin (p < 0.15), IL-6 (p < 0.89), or TNF-α (p < 0.49) noted between groups.

### Energy levels and food cravings

**Figure 8:**
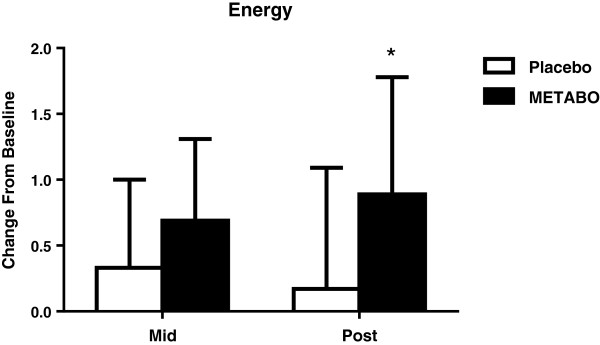
**Mean ± SD changes in energy levels, relative-to-baseline, in subjects who received METABO and placebo.** * indicates statistically significant difference (P < 0.05) between groups at the post time point via ANCOVA.

**Figure 9:**
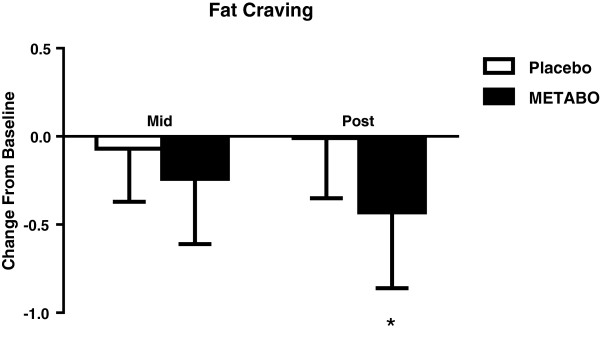
**Mean ± SD changes in fat cravings, relative-to-baseline, in subjects who received METABO and placebo.** * indicates statistically significant difference (P < 0.05) between groups at the post time point via ANCOVA.

Energy and food craving analyses from week 0 to week 8 are summarized in Table 5. Subjects who received METABO exhibited a statistically significant increase in relative energy levels (+ 29.3% versus +5.1%, respectively; p < 0.02, Figure 8). Subjects who received METABO also exhibited a statistically significant decrease in relative fats cravings compared to the placebo group (-13.9% versus -0.9%, respectively; p < 0.03, Figure 9). No statistically significant differences between the two groups were observed for sweet, fast food fats, carbohydrates or healthy food cravings.

**Table 5:** Relative energy and food craving analyses of METABO and placebo groups from week 0 through week 8

	**METABO**			**Placebo**			**P**
	**n = 27**			**n = 18**			**Value1**
	**Baseline**	**Mid point**	**End of study**	**Baseline**	**Mid point**	**End of study**	
	**(Week 0)**	**(Week 4)**	**(Week 8)**	**(Week 0)**	**(Week 4)**	**(Week 8)**	
Energy	3.04 ± 0.9	3.7 ± 0.67	3.93 ± 0.62	3.33 ± 0.69	3.67 ± 0.84	3.5 ± 0.92	0.22, 0.02*
Sweet	2.36 ± 0.8	2.05 ± 0.84	1.92 ± 0.91	2.48 ± 0.81	2.02 ± 0.84	2.12 ± 0.71	0.58, 0.30
FFF	2.85 ± 0.87	2.56 ± 0.94	2.35 ± 0.92	2.9 ± 0.54	2.28 ± 0.83	2.53 ± 0.68	0.48, 0.48
Fats	2.16 ± 0.85	1.92 ± 0.77	1.86 ± 0.8	2.04 ± 0.49	1.97 ± 0.46	2.02 ± 0.56	0.12, 0.03*
Carbs	2.26 ± 0.81	2.07 ± 0.74	2.01 ± 0.8	2.52 ± 0.64	2.1 ± 0.7	2.21 ± 0.61	0.86, 0.92
Healthy	2.44 ± 0.77	2.41 ± 0.72	2.38 ± 0.73	2.56 ± 0.49	2.21 ± 0.78	2.43 ± 0.51	0.42, 0.92

Values are mean ± SD.

^1^P values are for the differences between the two groups, METABO versus placebo at week 4 and week 8, respectively.

*Significant result via ANCOVA (i.e. week 8 time points are significantly different from each other after using the week 0 time point as the covariate).

*FFF*, fast food fats; Fats: total fats; Carbs: carbohydrates.

### Safety

No serious adverse events occurred during this study and analyses of standard clinical chemistry panels of serum and plasma revealed no statistically significant abnormalities of clinical importance. There were no significant between group effects for any cardiovascular variable during the 8-week trial, and the changes within groups were modest and non-significant. For resting systolic blood pressure, the placebo group went from 119.3 + 11.5 mmHg to 121.2 + 10.6 mmHg while the METABO™ group changed from 119.8 + 10.0 to 118.1 + 10.3 mmHg. Similarly, for resting diastolic blood pressure the placebo group dropped from 80.3 + 5.2 to 76.1 + 6.3 mmHg while the METABO™ group fell from 77.8 + 8.7 to 76.9 + 9.1 mmHg. For resting heart rate, the placebo group went from 69.4 + 8.4 to 69.9 + 7.9 beats/min while the METABO™ group did not experience a mean change (70.1 + 8.2 to 70.1 + 8.4 beats/min). The incidence of non-serious adverse events (e.g., stomach upset, etc.) were transient and similar, with no significant differences between placebo and METABO.

## Discussion

The results from this study demonstrate that as an adjunct to an 8-week diet and weight loss program, administration of METABO significantly decreases body weight, body fat mass, waist and hip girth, while increasing lean mass compared to the placebo. Although a restricted diet can lead to weight loss, it is often accompanied by a loss of lean tissue [28-30], which can have deleterious metabolic consequences. Because of this, it is important to achieve weight loss with a high ratio of fat to lean mass loss that is essential for both short-term efficacy and long-term metabolic health and body weight maintenance [31,32]. In this study, overweight subjects who received METABO achieved significantly greater improvements in their lean mass-to-fat mass ratio. Future studies should follow subjects during a washout period to determine if this effect helps maintain long-term weight control (i.e. minimize weight re-gain). Additionally, a future investigation should include a METABO only group with dietary control and no structured exercise program to explore the role of diet with METABO alone on body composition and metabolic outcomes.

Neither placebo nor METABO administration affected concentrations of blood lipids, including cholesterol, HDL, LDL, cholesterol/HDL ratio and TAG, although there was a strong trend (p < 0.07) for TAG concentrations to decrease more in the METABO group (-15.9%) compared to the placebo group (-2.6%). Future studies may attempt to explore this observation further with studies designed to look for differences in these important metabolic and biochemical markers as primary outcome measures.

Another important finding in our study relates to the observed differences in adipokine concentrations in the METABO group, although most of these did not achieve statistical significance. For example, we observed a trend for decreased serum resistin concentrations in subjects who received METABO compared to placebo at week 4, but not week 8. High serum resistin concentrations have been found in obese individuals and have been linked to insulin resistance, hence the trend for decreased resistin levels in METABO is an intriguing finding that requires further investigation in a future study [33]. The current study may have been underpowered to detect significant differences in serum adiponectin, given that fat loss occurred in both groups as a result of caloric restriction and a consistent exercise program. In addition, trends for maintaining elevated serum leptin (from week 0 to week 4) were observed in subjects who received METABO compared to placebo. Leptin acts on receptors in the hypothalamus to regulate appetite, energy expenditure, sympathetic tone and neuroendocrine function, and circulating levels have been shown to decline in response to caloric restriction or negative energy balance [34]. Leptin deficiency has been shown to promote hunger and food seeking behaviour, in addition to reduced metabolic rate in humans [35]. Collectively, the trend for resistin and significant change in leptin may help to partly explain the effects of METABO on body composition.

The combination of ingredients with potentially complementary and interactive mechanisms of action may account for the favorable changes observed in many of the clinical endpoints in the METABO group. Razberi-K® contains Raspberry ketone (4-(4-hydroxyphenyl) butan-2-one), which is a naturally occurring phenolic compound in red raspberry (*Rubus ideas*) that has been shown to enhance norepinephrine-induced lipolysis in adipocytes, prevent high-fat diet-induced body weight gain in mice, and increase adiponectin gene expression and secretion in adipocytes in culture [12,13]. Moreover, Wang et al. demonstrated anti-inflammatory benefits, improved antioxidant capacity, and enhanced leptin and insulin sensitivity in Sprague-Dawly rats using a high-fat diet induced nonalcoholic steatohepatitis (NASH) model [36]. From the limited preclinical literature, it appears that raspberry ketones require norepinephrine for maximizing their hormone-sensitive lipolytic action. Capsimax® is a concentrated capsicum extract found in an encapsulated beadlet form to decrease gastric irritation. Capsaicinoids have been shown in animal studies to activate TRPV1 receptors in vagal afferents of the gut, leading to sympathomimetic action with reductions in abdominal/visceral fat [37]. There have been a number of short-term human clinical studies utilizing between 2 mg/day and 10 mg/day of active capsaicinoids that have reproduced some of these preclinical animal efficacy and human clinical studies [37-39] including increases in norepinephrine secretion [15,17]. Further, a systematic review of 90 clinical trials, 20 of which were selected for inclusion demonstrated that capsaicinoid consumption of greater than 2 mg/day resulted in increases in energy expenditure of approximately 50 kcal/day and concentrations of anorexigenic hormone glucagon-like peptide-1 [37,39]. Moreover, significant decreases in energy intake of up to 8%, reductions in preoccupation with food and desire for fatty foods have been reported [39] that appears consistent with our food craving analyses in the METABO group (Table 5).

Advantra Z® is an ingredient extracted from the Citrus aurantium (traditional Chinese herb known as zhi-shi) and standardized for the bioactive alkaloid p-synephrine. Other alkaloids are present in the extract including: octopamine, hordenine, and n-methyltyramine. Taken together, the bioactive amines found in Advantra Z® have been shown to increase thermogenesis, and there is cell and tissue culture evidence to suggest lipolysis is accelerated via a β3 adrenergic receptor pathway [40]. A recent systematic review of human clinical studies involving Citrus aurantium with its primary p-synephrine alkaloid alone or in combination with other ingredients revealed reliable increases in resting metabolic rate of between 2.41% and greater than 7.2%, energy expenditure of up to 13.4%, and weight loss of over 2.9 kg, with no serious adverse events affecting hemodynamic, electrocardiographic, hematologic or clinical chemistry biomarkers when administered over the course of 6-12 weeks [22].

Caffeine is regarded as one of the most commonly consumed methylxanthine alkaloids known to act as an adenosine receptor antagonist and phosphodiesterase inhibitor. As such, the presence of caffeine may have contributed to amplifying the beta-adrenergic and lipolytic effects of the METABO formulation. Despite being on a calorie-reduced diet, subjects in this study reported feeling improved energy and decrease in cravings for energy-dense, fatty foods. Previous studies have indicated that food cravings are significantly related to food intake with specific cravings correlating with types of food consumed [24] and a high-fat diet is a strong risk factor for the development of obesity and metabolic syndrome, as a result of increased energy density and overall caloric intake [41]. Caffeine, in isolation or in combination with other bioactive nutritional compounds, has also been shown in multiple human clinical trials to increase the perception of energy, blunt appetite, and improve measures of mood, alertness, attention, and concentration [14,42,43]. Caffeine may be a thermogenic potentiator in METABO, as it has been shown to increase energy expenditure by 4-5% and fat oxidation by 10-16%, in addition to enhancing endurance and high-intensity exercise performance [44,45].

Although subject demographics were similar between groups, there was greater attrition of the placebo group relative to METABO. Most of the attrition was the result of poor compliance with the diet, supplement and/or exercise program. It has been reported that decreased levels of mental and physical energy and increased cravings for energy-dense foods can diminish dietary and exercise adherence during outpatient weight loss programs [46,47]. A notable finding in this regard is that, compared to the placebo group, the METABO group experienced a significant increase in their energy levels and decreased cravings for energy-dense foods. Future studies may examine if METABO improves adherence to a comprehensive diet and exercise weight loss program. Gender differences were not explored in our study, but future investigations are currently underway in an attempt to answer this question.

The authors would like to clarify why the data presented in Table 3 does not appear to underfeed each subject by 500 kcals/day. The mean target caloric intake for the METABO group using the Mifflin-St. Jeor equation multiplied by an activity factor of 1.2 –(minus) 500 kcal equals 1955 kcal/day. The target intake for placebo using same method was 1907 kcal/day. We realize these targets are greater than the mean of each group’s reported baseline caloric intake based on three-day food records. However, three-day food records are notorious for recall bias and an underestimation of actual energy consumption [48]. Thus, it is not surprising that both groups moved closer to their “target” kcal/day intake over the course of this 8 week study. The target caloric intakes being greater than the reported intakes from baseline (pre-intervention) three-day food records helps to explain why both groups may have actually increased their reported intakes by 4% and 9% for METABO and placebo, respectively. Therefore, it is plausible that subjects in both groups may have experienced even more dramatic body composition and/or metabolic benefits had the 500 kcal deficit been applied to their baseline, reported intakes as opposed to their estimated caloric requirements obtained via Mifflin-St. Jeor equation.

The obese and overweight state is characterized by chronic, low-grade systemic inflammation as a result of the expanded white adipose tissue compartment, particularly the visceral adipose depot. Adipose tissue from obese individuals is known to be an important endocrine organ capable of contributing to insulin resistance, persistent inflammation, and metabolic and vascular dysfunction via the perturbed adipokine secretion profile [34]. The collective action of garlic extract standardized for organosulfur compounds, ginger extract standardized for gingerols and shogaols, biotin and chromium in METABO may contribute to antiadipogenic, anti-inflammatory actions in conjunction with metabolic health benefits [20,21,36,37,49-51]. The bioactive compounds in garlic, ginger, and raspberry in addition to biotin and chromium have been suggested to modulate high-leverage metabolic pathways with nutrigenomic signaling, including: NF-kB, PPAR-γ, PPAR-α, orexigens, and aforementioned adipocytokines. It is conceivable that although increased sympathomimetic drive, lipolysis and thermogenesis contributed to the positive outcomes in body composition, the interaction of reduced dietary energy intake with exercise and METABO lead to further improvements in the adipokine profile that facilitated improvements in serum triacylglycerol, selective fat loss, skeletal muscle retention and abdominal girth reduction. It would be helpful for future studies to explore the influence of METABO on the systemic adipokine profile to clarify if this is one potential mechanism.

## Conclusion

In recent years, there have been numerous natural products being marketed and sold that claim to contain the right combination of vitamins, herbs and foods that can help with weight loss. However, very few of these products undergo finished product-specific research demonstrating their efficacy and safety. In the current study, as an adjunct to an 8-week diet and weight loss program, METABO administration augmented beneficial changes in body composition and anthropometric variables (hip and waist girth) in overweight men and women, and led to additional benefits on energy levels and food cravings. The placebo group had noticeable beneficial changes in body fat and non-significant improvements in certain metabolic variables as a result of diet and exercise alone, albeit these changes were less robust than in METABO group. METABO was safe and well-tolerated in all subjects, no serious adverse events were recorded, nor were differences in systemic hemodynamics or clinical blood chemistries observed between the two groups. Further studies are required to clarify the mechanisms by which METABO exerts its weight loss effects and its possible role in regulating adipokine concentrations.

## Competing interests

HLL and TNZ have received research funding and/or acted as consultants to raw material suppliers, nutraceutical and dietary supplement companies, including Ultimate Wellness Systems Inc, and Integrity Nutraceuticals Inc.

## Author’s contributions

HLL and TNZ contributed to the design and coordination of the study, drafting the manuscript, as well as oversight of data collection and analyses. JEH and SMH carried out the practical aspects of the study, including data collection and dietary analyses. SMA participated in the adipokine analyses and assisted in manuscript preparation. JPW performed the statistical analyses. AAF assisted in analysis and interpretation of data, as well as manuscript preparation. All authors participated in editing and approved the final draft of the manuscript.
